# Development of necroptosis-related gene signature to predict the prognosis of colon adenocarcinoma

**DOI:** 10.3389/fgene.2022.1051800

**Published:** 2022-10-24

**Authors:** Miaomiao Li, Tianyang Zhang, Wei Chen

**Affiliations:** ^1^ School of Life Sciences, North China University of Science and Technology, Tangshan, China; ^2^ Innovative Institute of Chinese Medicine and Pharmacy, Chengdu University of Traditional Chinese Medicine, Chengdu, China

**Keywords:** colon adenocarcinoma, necroptosis, gene signature, survival analysis, nomogram, molecular docking

## Abstract

Colon adenocarcinoma (COAD) is a common malignancy and has a high mortality rate. However, the current tumor node metastasis (TNM) staging system is inadequate for prognostic assessment of COAD patients. Therefore, there is an urgent need to identify reliable biomarkers for the prognosis COAD patients. The aberrant expression of necroptosis-related genes (NRGs) is reported to be associated with tumorigenesis and metastasis. In the present work, we compared the expression profiles of NRGs between COAD patients and normal individuals. Based on seven differentially expressed NRGs, a risk score was defined to predict the prognosis of COAD patients. The validation results from both training and independent external cohorts demonstrated that the risk score is able to distinguish the high and low risk COAD patients with higher accuracies, and is independent of the other clinical factors. To facilitate its clinical use, by integrating the proposed risk score, a nomogram was built to predict the risk of individual COAD patients. The C-index of the nomogram is 0.75, indicating the reliability of the nomogram in predicting survival rates. Furthermore, two candidate drugs, namely dapsone and xanthohumol, were screed out and validated by molecular docking, which hold the potential for the treatment of COAD. These results will provide novel clues for the diagnosis and treatment of COAD.

## 1 Introduction

Colon adenocarcinoma (COAD) is one of the most common cancers worldwide and the second leading cause of cancer death (Bray et al., 2018). Surgery and chemotherapy remain the mainstay of colon cancer treatment (Miller et al., 2019). At present, the prognostic assessment and treatment planning of COAD patients depend largely on the TNM staging system (Kehoe and Khatri, 2006). Even at the same tumor stage, however, due to tumor heterogeneity, there are still significant disparities in disease progression and clinical outcomes. Hence, TNM staging system is not fully capable of predicting the prognosis of colon cancer patients. Accordingly, more reliable prognostic biomarkers are needed for the diagnose of colon cancer. The occurrence of tumors is inseparable from the abnormal gene expressions, and which have been used as biomarkers to predict the prognosis of diseases (Liu et al., 2018; Gao et al., 2020). Most recently, it was reported that the aberrant expression of necroptosis-related genes (NRGs) is closely associated with the tumorigenesis and metastasis (Ding et al., 2022; Qi et al., 2022).

Necroptosis is a double-edged sword in the carcinogenesis and progression of cancer. The tumor cell necrosis can lead to tumor necrosis and promoted tumor metastasis (Lebrec et al., 2015). For example, the pro-necrosis proteins, such as RIPK1, RIPK3, and MLKL, play key roles in promoting tumor growth (Liu et al., 2016). Conversely, necroptosis also exhibits tumor suppressive effects. Results from two independent groups showed that overexpression of the cell necroptosis factor RIP3 inhibited the proliferation of colon cancer cells (Feng et al., 2015; Krysko et al., 2017). These findings show that cellular necrosis has a multifaceted biological role in carcinogenesis and invasion. Therefore, NRGs have gained attentions of researchers and have been proposed for risk classification and survival prediction of COAD patients. For example, Huang *et al.* found that a necroptosis-related miRNA risk signature consisting of seven miRNAs could be used to predict the prognosis of colon cancer patients (Huang et al., 2021). Subsequently, Yang et al. constructed a necroptosis-related miRNA signature for predicting colon cancer prognosis (Yang et al., 2022). Later on, Liu *et al.* proposed another model to predict the prognosis of colon cancer patients based on necroptosis-related lncRNAs(Liu et al., 2022). However, these studies only used the TCGA dataset for internal validation, and did not test their results on the external validation dataset. Moreover, their accuracies for predicting the prognosis of colon cancer patients are not satisfactory. Therefore, new reliable signatures are needed to predict survival in COAD patients.

**FIGURE 1:**
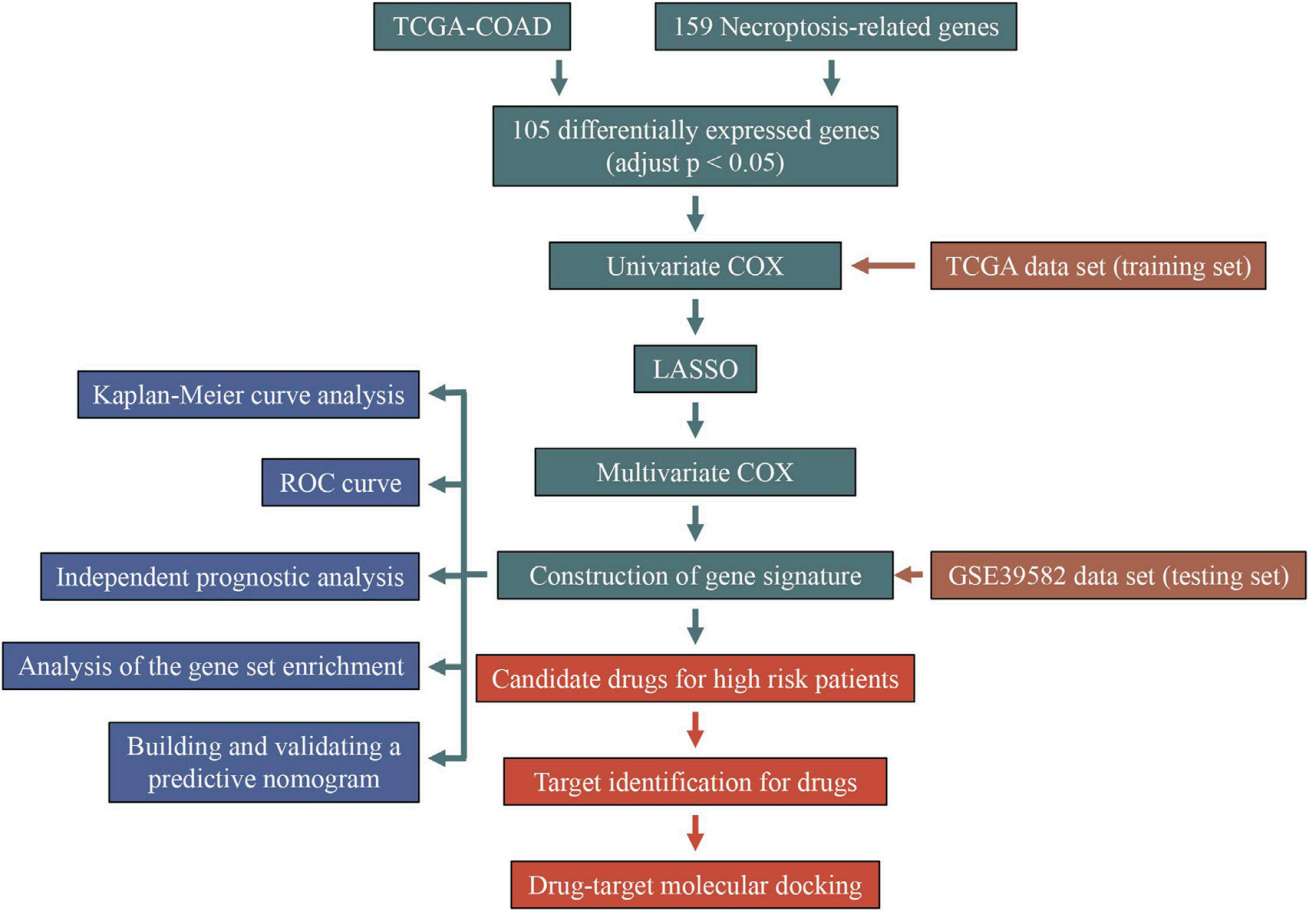
The flow chart of this study.

In this study, based on the seven differently expressed NRGs, we proposed a new NRGs-based model to predict the prognosis of COAD patients. The proposed model is able to distinguish the high and low risk patients in both internal training and external testing dataset with higher accuracies. In order to facilitate its clinical use, a prognostic nomogram was built to quantify the death risk of individual patients. Moreover, on the basis of Connectivity Map (Cmap) database (Subramanian et al., 2017), the candidate drugs for the treatment of high risk patients were screened out and validated by molecular docking analysis. The workflow of this work was shown in Figure 1.

## 2 Materials and methods

### 2.1 Data collection

The TCGA public database (https://portal.gdc.cancer.gov/) was used to gather COAD RNA-sequencing (RNA-seq) data and clinical follow-up information. After excluding the samples with a follow-up period of less than 30 days and samples with duplicate patients, we obtained 417 tumor tissue samples and 41 non-tumor tissue samples. The RNA-seq data were then converted to transcripts per million (TPM). The 556 independent validation samples were fetched from the GEO dataset (https://www.ncbi.nlm.nih.gov/geo/) with the accession number GSE39582.

### 2.2 Acquisition of differentially expressed NRGs

159 NRGs involved in the necroptosis signaling pathway were obtained from the KEGG database (https://www.genome.jp/kegg/, Supplementary Table S1). The limma package (version 3.42.2) in R software (version 3.6.1) was used to perform the differential expression analysis of NRGs in tumor and non-tumor tissue with *p* < 0.05, false discovery rate (FDR) < 0.05 and |log2FoldChange|>0. The pheatmap (version 1.0.12) and EnhancedVolcano (version 1.4.0) packages were used for the visualization of differentially expressed genes (DEGs). The R package clusterProfiler (Yu et al., 2012) (version 3.14.3) was used for GO and KEGG enrichment analysis, and enrichplot (version 1.6.1) was used for visualization studies.

### 2.3 Definition of the NRGs based risk score

Univariate Cox regression analysis was used to screen NRGs that were significantly (*p* < 0.05) associated with COAD survival rates. And then, a LASSO-Cox regression analysis was used to select the NRGs signature. The genes thus obtained were used to define a risk score defined as following,

$$Risk\;score=\overset{n}{\underset{i=1}{\sum }}{Coef}_{i}*{\mathrm{Exp}}_{i}$$

where *i* stands for one of the *n* NRGs, Exp_*i*_ is the expression level of gene *i*, and Coef_*i*_ is the corresponding coefficient determined by LASSO-Cox regression analysis. Patients were then split into two subgroups, namely low risk group and high risk group, based on the median of risk score. The survival (version: 3.2–7) and glmnet (version: 4.1–1) (Friedman et al., 2010) packages in R were used for the analysis.

### 2.4 Prognostic performance analysis of risk signature

Kaplan-Meier survival analysis was used to assess the survival differences between the two risk groups. The receiver operating characteristic (ROC) curve was used to evaluate the accuracy for predicting the overall survival (OS) of COAD patients. The univariate and multivariate Cox regression analysis were used to test whether the risk score is independent of the other clinical traits (age, sex, stage, TNM grade).

### 2.5 Gene set enrichment analysis

The org. Hs.eg.db (version 3.10.0), clusterProfiler (version 3.14.3), and ggplot2 (version 3.3.3) packages in R were used to perform gene set enrichment analysis (adjust *p* < 0.05).

### 2.6 Construction and verification of nomogram

For facilitating clinical use, the nomogram was built by using the rms (version 6.1–1) and survival (version 3.2–7) packages in R. The discriminative ability of the nomogram was assessed by using AUC smoothing curve and C-index. Calibration curves were used to evaluate the relationship between actual results (45-degree diagonal) and predictive probabilities. The accuracy was obtained after 1,000 times of bootstraps (Huang et al., 2016).

### 2.7 Candidate drug identification

The Cmap database was used to identify the drugs for the treatment of patients in the high risk group. The DEGs between high and low risk groups in the TCGA-COAD cohort were identified by using differential expression analysis (|log_2_FC|≥1.5, *p* < 0.05, and FDR<0.05). By inputting the DEGs of the high risk group into Cmap, the potential drug candidates were obtained and sorted based on their scores ranging from -100 to 100. The positive scores indicate the synergistic effects of the drugs on diseases, while negative scores indicate antagonistic effects of the drugs on diseases (Subramanian et al., 2017). Hence, the drugs with negative scores hold the potential for the treatment of diseases. In the present work, drugs with score less than -80 were selected out for further analysis.

### 2.8 Drug targets identification and validation

The targets of the candidate drugs were predicted by using the STITCH database (http://stitch.embl.de/) with the confidence score greater than 0.8 (Szklarczyk et al., 2016). Only the targets that differentially expressed between high and low risk groups and significantly correlated with patient OS were screened out. The 2D structures of candidate drugs were taken from the PubChem database (https://pubchem.ncbi.nlm.nih.gov/), and their 3D chemical structures were drawn using ChemOffice 2019. The protein structures of the targets were obtained from the RCSB PDB database (PDB, http://www.pdb.org/). The AutoDockTools-1.5.6 and Autodock Vina-1.1.2 were used to perform molecular docking between candidate drugs and the targets (Morris et al., 2009; Trott and Olson, 2010). A docking free energy less than -5.0 kcal/mol was regarded as a stable binding (Li et al., 2022). PyMOL-2.4.0 and Discovery studio 4.5 were used to visualize the molecular docking results.

### 2.9 Statistical analysis

All statistical analysis and result visualization were performed by using R (version 3.6.1). The Wilcoxon test was utilized to determine the difference between the two groups. The Pearson correlation coefficient was calculated to assess the associations between clinicopathological characteristics and risk scores. *p* < 0.05 was regarded as statistically significant for two-sided tests.

## 3 Results

### 3.1 Differentially expression of NRGs

**FIGURE 2:**
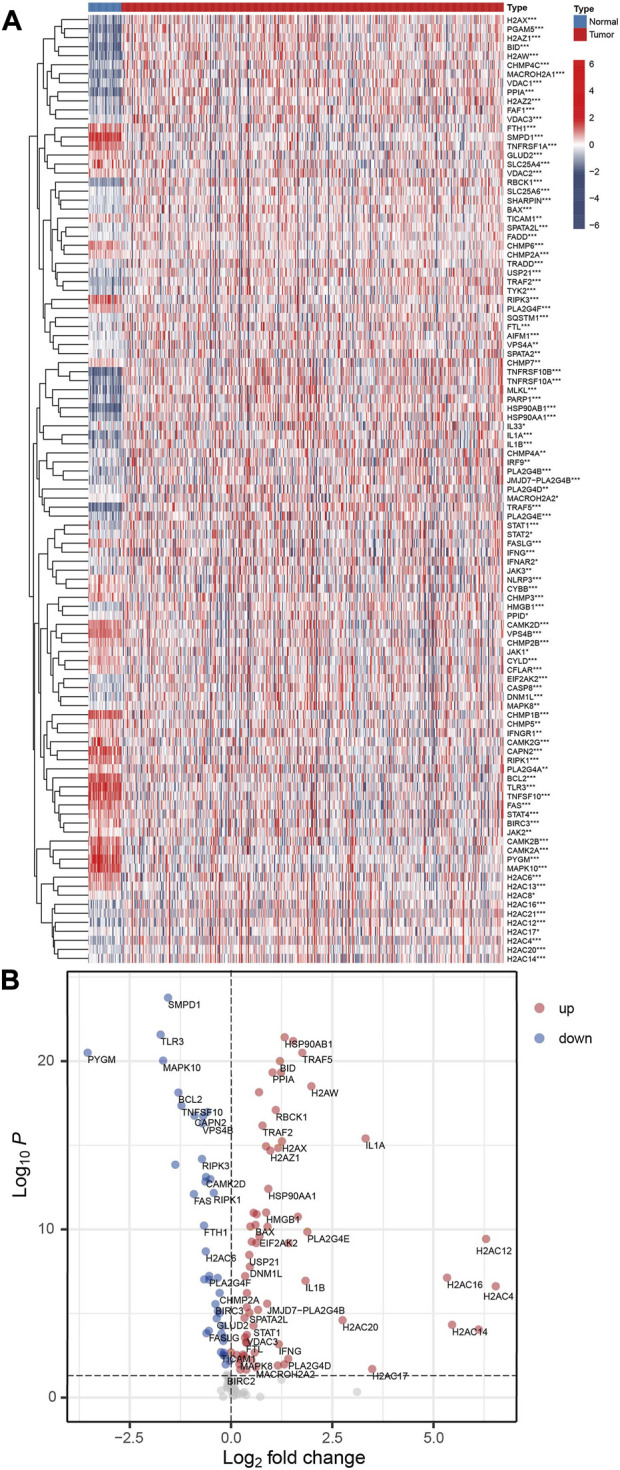
The differentially expressed NRGs. **(A)** Heatmap for the 105 differentially expressed NRGs. Red is tumor tissue samples, and blue is normal samples (*p < 0.05; **p < 0.01; ***p < 0.001). **(B)** A volcano plot of NRGs. Up-regulated and down-regulated genes are indicated by red and blue, respectively.

Among the 159 NRGs, 105 were differentially expressed (*p* < 0.05 and FDR<0.05) between normal and COAD samples, Figure 2A. Further analysis demonstrated that 40 NRGs were significantly under-expressed in tumor tissues, and 65 were significantly over-expressed, Figure 2B and Supplementary Table S2. The results from KEGG analysis demonstrated that the most significantly enriched pathway of the differentially expressed NRGs is necroptosis (Supplementary Figures S1A,B). However, the GO enrichment analysis demonstrated that the under-expressed and over-expressed NRGs were enriched in different entries (Supplementary Figure S1C and S1D). For the biological process (BP), the up-regulated genes were most significantly enriched in regulation of apoptotic signaling pathway, while down-regulated genes were in necroptotic process. In terms of cellular component (CC), the up-regulated genes were in nuclear chromatin, while down-regulated genes were in endosome membrane. The most significantly enriched molecular function of up-regulated genes is cytokine receptor binding, while that of down-regulated genes is protein serine/threonine kinase activity. These results demonstrated that the differentially expressed NRGs were associated distinct biological functions.

### 3.2 Establishment and validation of the prognostic NRGs signature in COAD patients

Univariate Cox regression analysis showed that eight NRGs were significantly associated with the survival status of COAD patients (Supplementary Table S3). We further employed the LASSO-Cox regression analysis to assess the survival rates of COAD patients, and obtained seven NRGs (Supplementary Figures S2A,B, Supplementary Table S4). It was found that five of them (CAMK2B, H2AC6, MLKL, RBCK1, and TRAF2) were risk factors and two (RIPK3 and VDAC3) were protective factors (Supplementary Figure S2C). Then, they were used to build the prognostic-related NRG signature (also called risk score, see section 2.3).

**FIGURE 3:**
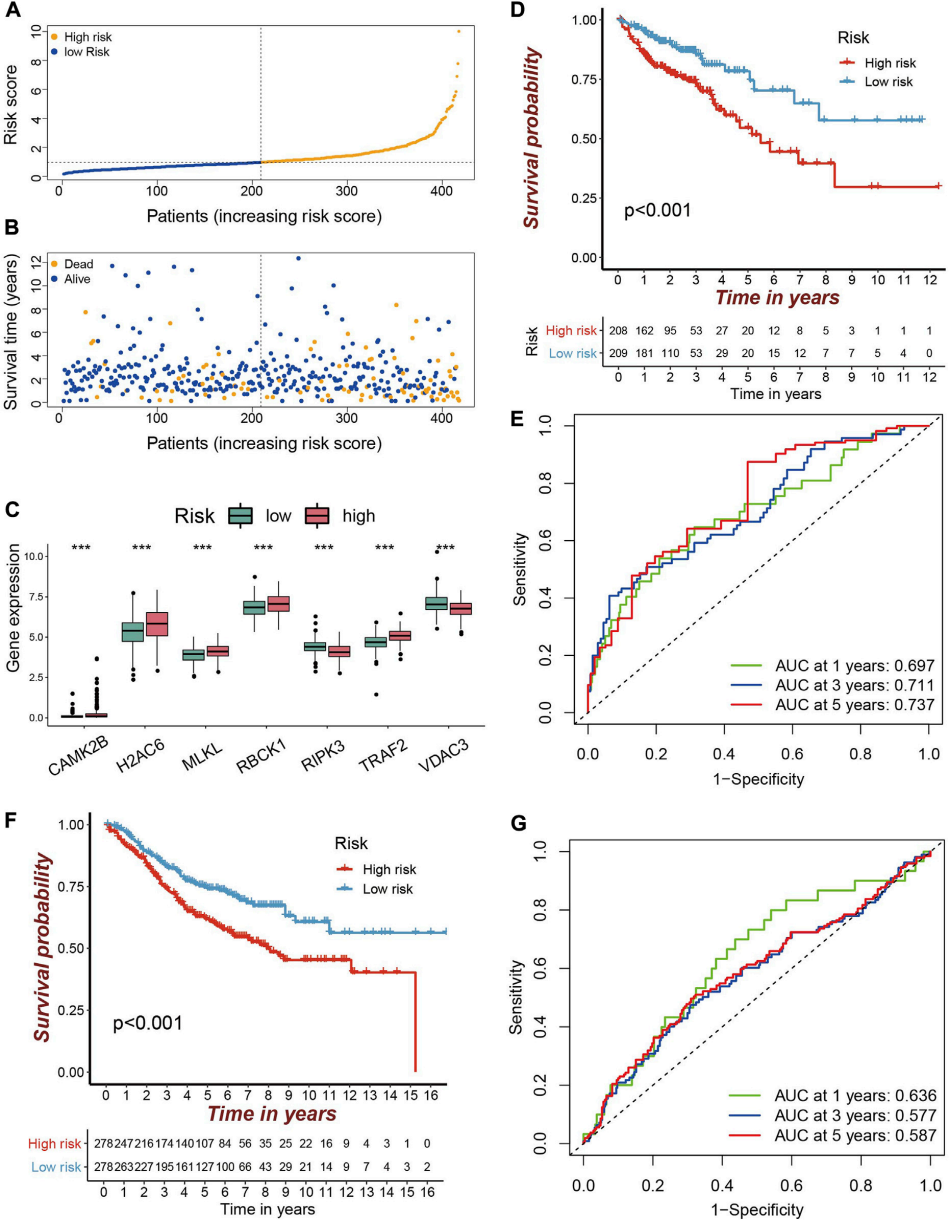
Validation of the prognostic NRGs signature in COAD patients **(A–B)** The TCGA-COAD samples were divided into high and low risk groups according to the median risk score. The larger the risk score, the more the samples of deaths. **(C)** Differentially expression of prognostic genes in high and low risk groups are depicted in a boxplot. Red is the high risk group and green is the low risk group (***p < 0.001). **(D)** Kaplan-Meier curve for predicting OS in the TCGA cohort. Red is the high risk group and blue is the low risk group. **(E)** ROC curve in the TCGA cohort. **(F)** Kaplan–Meier curve for predicting OS in the GEO cohort. **(G)** ROC curve in the GEO cohort.

On the basis of the prognostic-related NRG signature, each sample was assigned a risk score. With the median risk score as a cut-off value, the samples in the dataset were divided into high risk group (n = 208) and low risk (n = 209) group, respectively. With the increasement of risk score, the number of deaths increased progressively (Figures 3A,B). In the high risk group, the risk factors were significantly overexpressed, while the protective factors were significantly under expressed (Figure 3C). The Kaplan-Meier survival curve based on the risk score shows that the high and low risk groups have significantly different survival rates. Patients in the high risk group having a lower OS than those in the low risk group (Figure 3D).

The performance of the risk score for predicting the patient’s OS was evaluated by using the ROC curve. Its area under the ROC curve (AUC) for 1-year, 3-year and 5-year OS were 0.697, 0.711, and 0.737 (Figure 3E), respectively. The AUC for predicting 5-year OS is better than those reported by Huang et al. (AUC = 0.724) (Huang et al., 2021), Yang *et al.* (AUC = 0.656) (Yang et al., 2022), and Liu *et al.* (AUC = 0.639) (Liu et al., 2022). The 7-NRGs based risk score model was further validated in the independent GEO dataset (GSE39582). Compared with low risk patients, patients in the high risk group also had a worse OS (Figure 3F). The AUCs for 1-, 3-, and 5-year OS were 0.636, 0.577, and 0.587 (Figure 3G). These results indicate that the developed prognostic model is reliable, and the seven NRGs holds the potential to be efficient biomarkers for the prognosis of COAD.

### 3.3 NRGs signature is an independent prognostic factor

**FIGURE 4:**
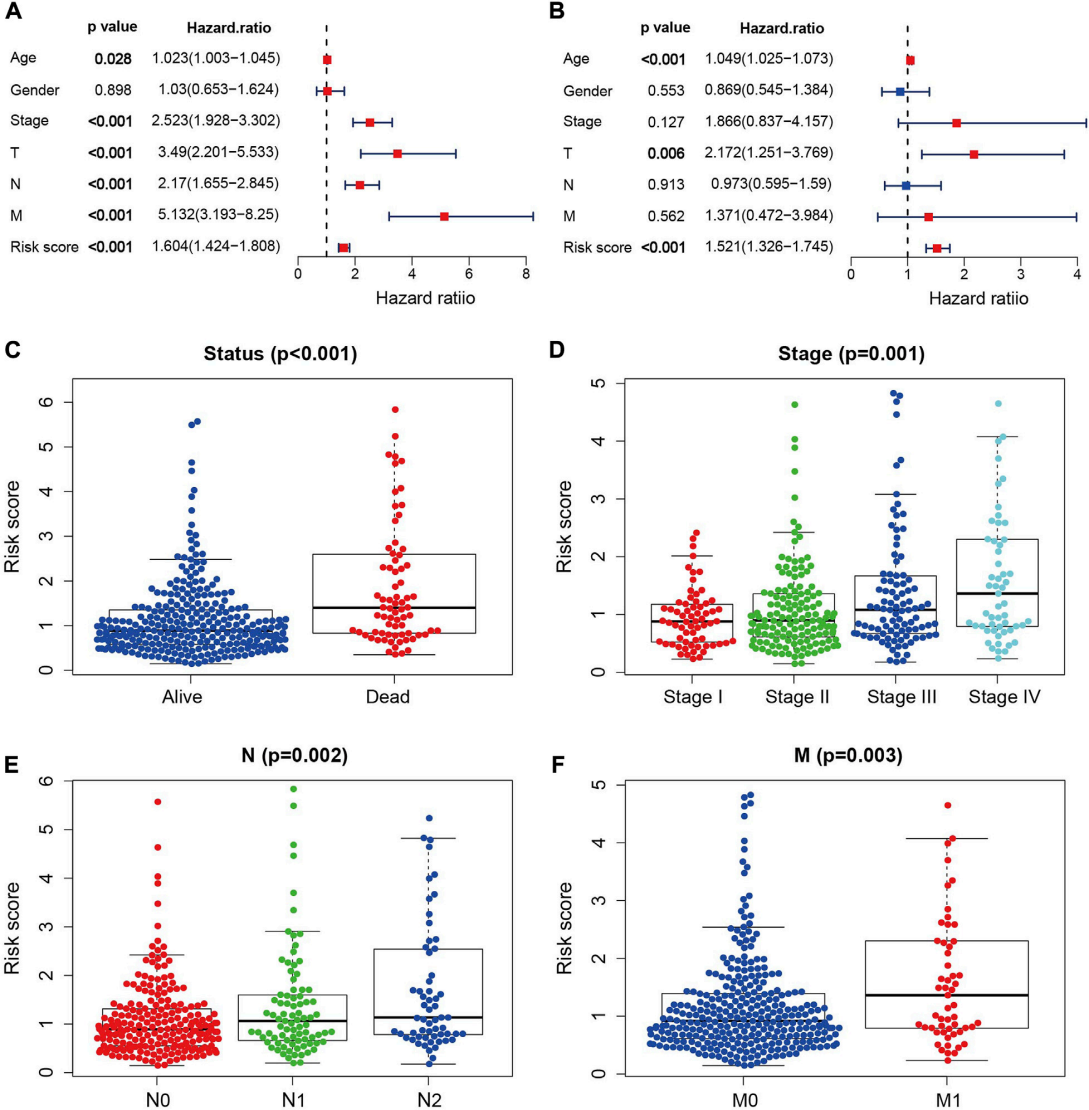
Independent prognostic analysis. **(A)** Univariate independent prognostic analysis in the TCGA cohort. **(B)** Multivariate independent prognostic analysis in the TCGA cohort. **(C–F)** NRGs signature based outcome stratification of different clinicopathological features (Status, Stage, N, M).

The univariate and multivariate Cox regression analysis were further performed to test whether the risk score is independent of the other clinical factors. The result of univariate Cox regression analysis demonstrated that risk score, age, stage, T, N, and M stages were all associated with patient survivals (Figure 4A). The multivariate Cox regression analysis demonstrated that the risk score is independent of the above mentioned clinical factors (Figure 4B), and can satisfactorily classify the survival status, tumor stage, N and M grades of COAD patients (Figures 4C–F). With the increase of the risk score, the pathological degree of tumor become worse. These findings imply that the risk score is effective in predicting the survival and prognosis of COAD patients.

### 3.4 Gene set enrichment analysis

**FIGURE 5:**
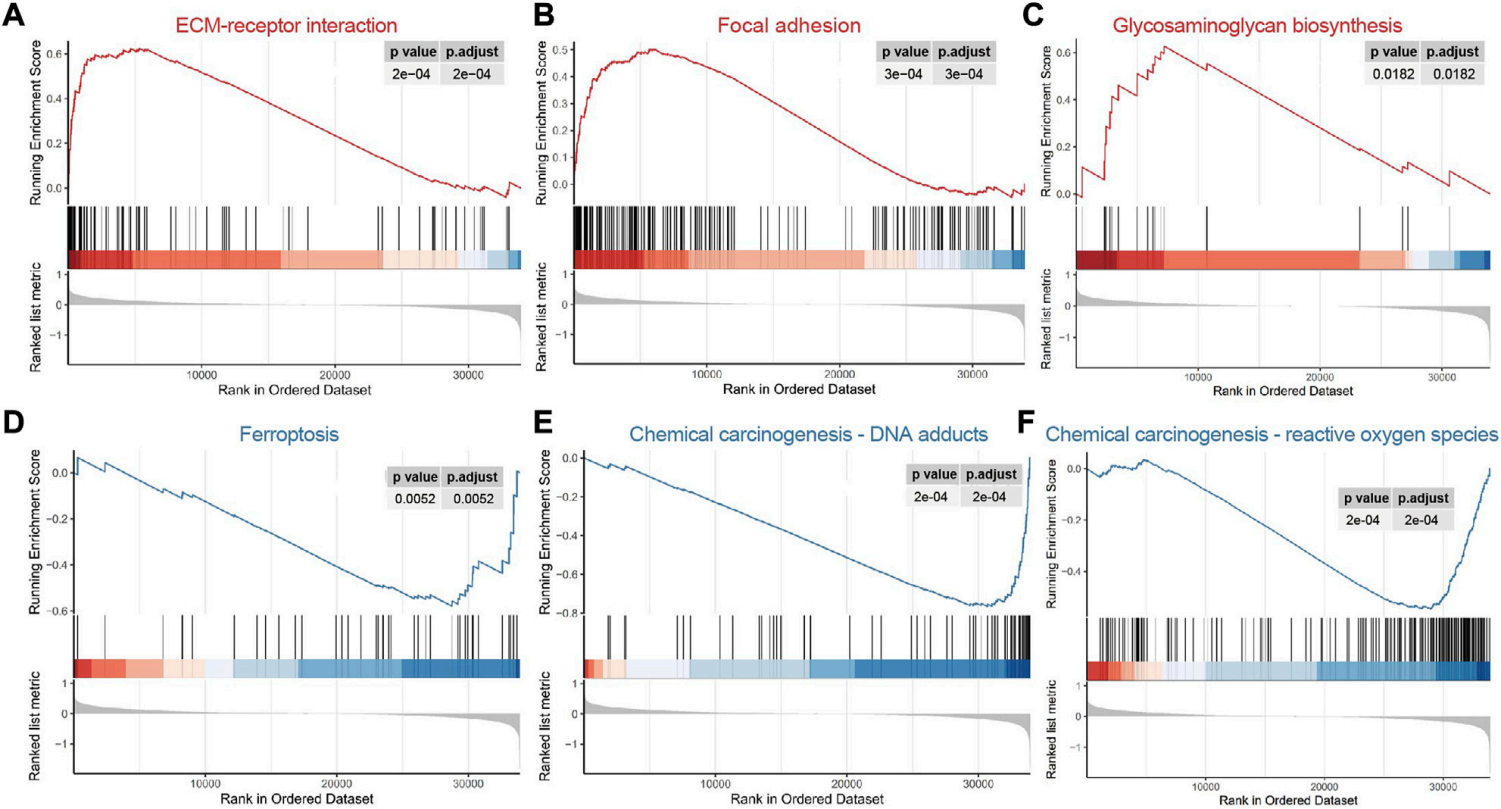
Functional gene set enrichment analysis. **(A–C)** The pathways enriched in high risk group; **(D–F)** The pathways enriched in low risk group.

The results of GSEA demonstrated that the focal adhesion, ECM-receptor interaction and glycosaminoglycan biosynthesis pathways were enriched in the high risk group (Figures 5A–C, Supplementary Table S5), indicating that the tumor metastasis and invasion were the characteristics of high risk group. Chemical carcinogenesis-DNA adducts, ferroptosis and chemical carcinogenesis-reactive oxygen species were the enriched pathways of the low risk group (Figures 5D–F), demonstrating that tumor formation and progression are the characteristics of the low risk group. These results were consistent with the progression of COAD.

### 3.5 Construction and evaluation of a prognostic nomogram for individual COAD patients

**FIGURE 6:**
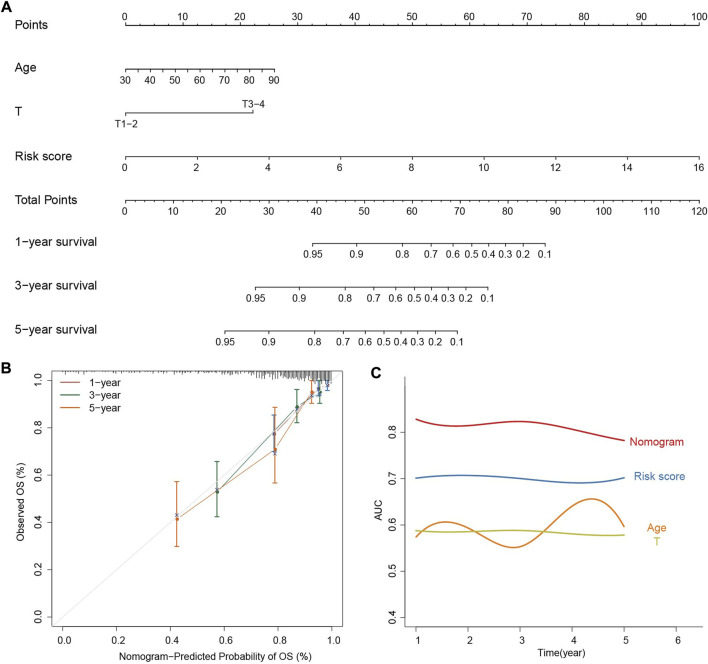
Construction and evaluation of the prognostic nomogram. **(A)** The nomogram predicts the probability of the 1-, 3-, and 5-year OS. **(B)** The calibration plot of the nomogram for predicting the probability of the 1-, 3-, and 5-year OS. **(C)** AUC smooth curve for evaluating the accuracy of nomogram predictions.

In order to facilitate personalized survival prediction of COAD patients, the nomogram was built based on risk score, T and age (Figure 6A). The C-index and AUC were used to evaluate the performance of the nomogram, and the calibration curve is used to see how well the prediction matches the actual. The C-index of the model is 0.75 and the 1-, 3-, and 5-year survival probabilities are quite close to ideal performance (45-degree line), indicating satisfactory performance of the nomogram in predicting OS (Figure 6B). When compared with a single kind of prognostic feature, the nomogram outperforms risk score, T and age for predicting the survivals of COAD patients, suggesting the better performance of nomogram (Figure 6C).

### 3.6 Candidate drugs identification for high risk COAD patients

To identify potential drugs for the treatment of high risk COAD patients, a total of 237 DEGs (Supplementary Table S6) were used as the inputs of the Cmap database, among which, 210 DEGs were significantly up-regulated and 27 were significantly down-regulated in the high risk group. It was found that five drugs, namely MST-312, flucytosine, ganglioside, xanthohumol and dapsone, were with the scores less than -80 and held the potential for the treatment of high risk patients (Table 1).

**TABLE 1:** Summary of connectivity map prediction results.

Drugs	Score	Description
MST-312	-93.38	Telomerase inhibitor
Flucytosine	-87.35	Antifungal
Ganglioside	-85.85	SRC activator
Xanthohumol	-82.18	ATPase inhibitor
Dapsone	-80.21	Bacterial antifolate

### 3.7 Targets screening and molecular docking

**FIGURE 7:**
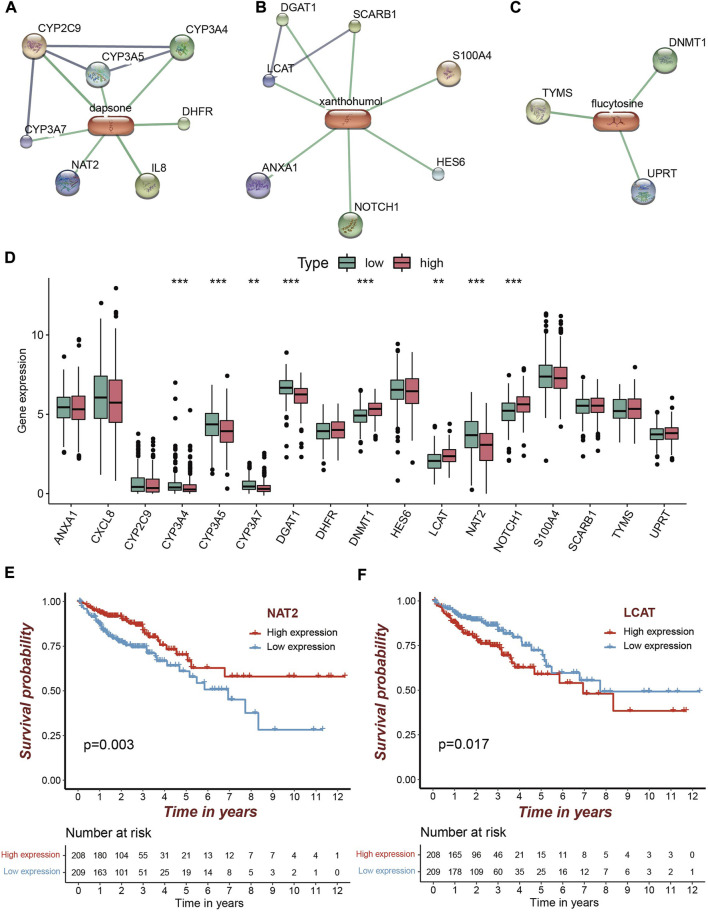
Candidate drugs screening for high risk patients and target identification. **(A–C)** The identified targets (confidence score>0.8) from STRING for dapsone, xanthohumol and flucytosine, respectively. **(D)** Eight of the 17 targets were significantly differentially expressed, of which five were significantly under-expressed in the high risk group and three were significantly over-expressed (**p < 0.01; ***p < 0.001). **(E,F)** Patients with a high NAT2 expression and patients with a low LCAT expression had a higher survival rate.

Based on the STITCH database, we obtained 17 targets for the five candicate drugs, including seven for dapsone, three for flucytosine, and seven for xanthohumol, respectively (Figures 7A–C). Eight of the 17 genes were differentially expressed in high risk group. NOTCH1, DNMT1, LCAT were over-expressed, while CYP3A4, NAT2, DGAT1, CYP3A5, CYP3A7 were under-expressed (Figure 7D). Further analysis demonstrated that only two of the eight differentially expressed genes were significantly associated with the survival of COAD patients (Figures 7E,F and Supplementary Figure S3). The patients with a high expression of NAT2 and a low expression of LCAT exhibit the higher survival rate (Figures 7E,F). Therefore, it is speculated that the drugs xanthohumol and dapsone may affect tumor progression by affecting the abnormally expression of LCAT and NAT2, respectively.

**FIGURE 8:**
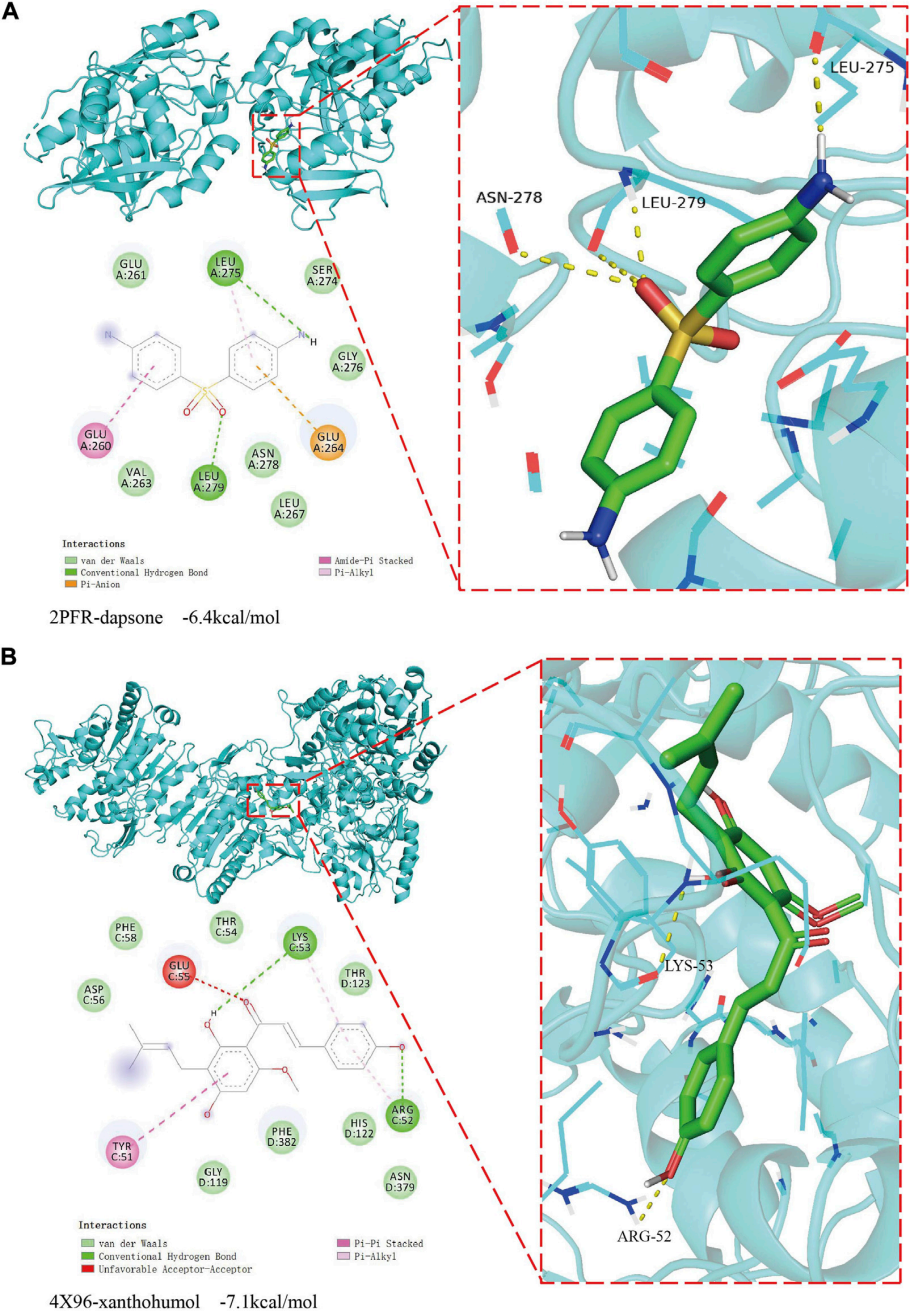
The result of molecular docking between candidate drugs and targets. **(A)** The molecular docking results between dapsone and its target NAT2. **(B)** The molecular docking results between xanthohumol and its target LCAT.

To validate whether the xanthohumol and dapsone could interact with target genes, the molecular docking was performed between the drugs and target genes, i.e. dapsone and NAT2, xanthohumol and LCAT, respectively. The dapsone and NAT2 (PDB ID: 2 P FR) had a docking affinity score of -6.4 kcal/mol (Figure 8A). Dapsone binds to NAT2 through interacting with amino acid residues, such as glu261, leu275, ser274, gly276, glu264, leu267, asn278, leu279, val263 and glu260. The docking affinity score between xanthohumol and LCAT (PDB ID: 4X96) was -7.1 kcal/mol (Figure 8B). Xanthohumol binds to LCAT through interacting with amino acid residues, such as asp56, phe58, glu55, thr54, lys53, thr123, arg52, asn379, his122, phe382, gly199 and tyr51. These results demonstrate that dapsone and xanthohumol possess good combination with their targets, and hold the potential to be the drugs for the treatment of COAD.

## 4 Discussion

The development of biomarkers and therapeutic targets at the molecular level is crucial for the prognosis and treatment of COAD. Tumorigenesis and metastasis are both aided by necroptosis (Stoll et al., 2017; Seehawer et al., 2018; Yan et al., 2022). Dysregulated expression of necroptosis genes can lead to chronic colonic inflammation which promotes colon cancer growth (Wang et al., 2020), suggesting that necroptosis is important for the development of COAD. At the meantime, it was also reported that medicines and substances that can interact with necroptosis genes have anticancer potentials (Su et al., 2015; Gong et al., 2019). In the present work, we therefore developed a NRGs based model for predicting the prognosis of COAD patients and identified the candidate drugs for the treatment COAD.

The proposed risk score model was built by using seven differentially expressed NRGs, namely CAMK2B, H2AC6, MLKL, RBCK1, VDAC3, RIPK3, and TRAF2. CAMK2B regulates the microenvironmental remodeling of renal papillary cell carcinoma, which has an anti-tumor effect (Jia et al., 2022). H2AC6, which belongs to the H2A family of histones, is a replication-dependent histone. Histone H2A has been linked to diabetic nephropathy, atherosclerosis, cardiovascular disease, and hypertensive kidney injury (Gao et al., 2013; Jiang et al., 2018; Yerra and Advani, 2018; Pei et al., 2021). MLKL may serve as a promising target to block tumor regeneration and participate in the regulation of necroptosis pathway, thereby improving the efficacy of radiation therapy for colorectal cancer (Wang et al., 2019). Overexpression of RBCK1 was reported to be associated with a poor prognosis in colorectal cancer patients (Liu et al., 2019). VDAC3 has been linked to cancer and pathology as a potential marker of mitochondrial status (Reina et al., 2016). Up-regulation of RIPK3 can prevent the development of liver cancer (Wu et al., 2020). TRAF2 is a tumor suppressor gene in colon cancer (Moon et al., 2021). Considering that RNA modifications were associated with the development of cancers, we performed the conservation analysis of N^6^-methyladenosine (m^6^A) modification for the seven genes by using ConsRM(Song et al., 2021). The conserved m^6^A sites were identified in TRAF2 and RBCK1, suggesting that m^6^A modification may be also associated with the pathogenesis of COAD.

Based on the proposed model, the patients in the TCGA cohort were clustered into low and high risk groups. In the high risk group, patients have a considerably shorter OS than those in the low risk group. The ROC curves obtained from the TCGA training data and the independent GEO data indicated that the proposed model has a relative high accuracy for predicting the OS of COAD patients and could be utilized as an independent predictor to predict patients’ risk of death.

The results from GSEA enrichment analysis demonstrated that the tumor metastasis and invasion associated signaling pathways were enriched in the high risk group (Figure 5). For example, the focal adhesion signaling pathway is closely related to tumor invasion (Golubovskaya and Cance, 2010). ECM-receptor interaction is an important pathway for colorectal cancer cell metastasis (Nersisyan et al., 2021). Glycosaminoglycan can promote cancer angiogenesis and metastasis (Wei et al., 2020). Signaling pathways related to tumor formation and progression were enhanced in the low risk group. Ferroptosis and chemical carcinogenesis promote the occurrence and development of cancer (de Bono et al., 2020; Chaudhary et al., 2021).

In order to provide insights for the treatment of COAD, we identified two candidate drugs, namely dapsone and xanthohumol, from the Cmap database. The dapsone improves the overall survival of colon cancer patients by inhibiting the expression level of tumor growth-driving elements IL-8 (Fisher et al., 2019; Kast et al., 2022). Xanthohumol acts as a carcinogenic inhibitor, low dose xanthohumol treatment blocks the proliferation and spread of primary colon cancer cells (Torrens-Mas et al., 2022). The results of molecular docking analysis demonstrated that dapsone and xanthohumol can interact with NAT2 and LCAT, respectively. Thus, dapsone and xanthohumol may alter the tumor progression of high risk COAD patients by acting on NAT2 and LCAT, respectively. Further experimental analysis was needed to illustrate the detail mechanisms.

Taken together, we developed a NRGs signature that can be used to predict the prognosis of COAD patients and screened out two candidate drugs for the treatment of high risk COAD patients. Inevitably, the following limitations should be considered in the future works. First, the robustness of the proposed model should be validated by large-scale prospective trials or cell experiments. Second, experiments are needed to validate the interactions between candidate drugs and targets and to demonstrate their treatment mechanisms on COAD. In addition, the data from the RNA modification databases, such as m6A-atlas (Tang et al., 2021), m5C-atlas (Ma et al., 2022), and m7Ghub (Song et al., 2020), should be integrated to further examine whether RNA modifications are associated with COAD as well.

## Data Availability

The datasets presented in this study can be found in online repositories. The names of the repository/repositories and accession number(s) can be found in the article/Supplementary Material.

## Abbreviations

AUC, area under the ROC curve; COAD, colon adenocarcinoma; Cmap, connectivity map; DEGs, differentially expressed genes; FC, fold change; GEO, gene expression omni molecular docking bus; GSEA, gene set enrichment Analysis; HR, hazard ratio; LASSO, least absolute shrinkage and selection operator; M, metastasis; N, node; NRGs, necroptosis-related genes; OS, overall survival; PDB, protein data bank; ROC, receiver operating characteristic; RCSB, Research Collaboratory for Structural Bioinformatics; T, tumor; TCGA, the cancer genome atlas; TPM, transcripts per million.

## References

[B1] Bray F. Ferlay J. Soerjomataram I. Siegel R. L. Torre L. A. Jemal A. (2018). Global cancer statistics 2018: GLOBOCAN estimates of incidence and mortality worldwide for 36 cancers in 185 countries. Ca. Cancer J. Clin. 68 (6), 394–424. 10.3322/caac.21492 30207593

[B2] Chaudhary N. Choudhary B. S. Shah S. G. Khapare N. Dwivedi N. Gaikwad A. (2021). Lipocalin 2 expression promotes tumor progression and therapy resistance by inhibiting ferroptosis in colorectal cancer. Int. J. Cancer 149 (7), 1495–1511. 10.1002/ijc.33711 34146401

[B3] de Bono J. S. Guo C. Gurel B. De Marzo A. M. Sfanos K. S. Mani R. S. (2020). Prostate carcinogenesis: Inflammatory storms. Nat. Rev. Cancer 20 (8), 455–469. 10.1038/s41568-020-0267-9 32546840

[B4] Ding C. Yu Z. Zhu J. Li X. Dai M. Qiang H. (2022). Construction and validation of a necroptosis-related gene signature for predicting prognosis and tumor microenvironment of pancreatic cancer. Dis. Markers 2022, 9737587. 10.1155/2022/9737587 35756487 PMC9214653

[B5] Feng X. Song Q. Yu A. Tang H. Peng Z. Wang X. (2015). Receptor-interacting protein kinase 3 is a predictor of survival and plays a tumor suppressive role in colorectal cancer. Neoplasma 62 (4), 592–601. 10.4149/neo_2015_071 25997957

[B6] Fisher R. C. Bellamkonda K. Alex Molina L. Xiang S. Liska D. Sarvestani S. K. (2019). Disrupting inflammation-associated CXCL8-CXCR1 signaling inhibits tumorigenicity initiated by sporadic- and colitis-colon cancer stem cells. Neoplasia 21 (3), 269–281. 10.1016/j.neo.2018.12.007 30738331 PMC6370871

[B7] Friedman J. Hastie T. Tibshirani R. (2010). Regularization paths for generalized linear models via coordinate descent. J. Stat. Softw. 33 (1), 1–22. 10.18637/jss.v033.i01 20808728 PMC2929880

[B8] Gao C. Chen G. Liu L. Li X. He J. Jiang L. (2013). Impact of high glucose and proteasome inhibitor MG132 on histone H2A and H2B ubiquitination in rat glomerular mesangial cells. J. Diabetes Res. 2013, 589474. 10.1155/2013/589474 23738337 PMC3657404

[B9] Gao T. Du T. Hu X. Dong X. Li L. Wang Y. (2020). Cosmc overexpression enhances malignancies in human colon cancer. J. Cell. Mol. Med. 24 (1), 362–370. 10.1111/jcmm.14740 31633299 PMC6933370

[B10] Golubovskaya V. M. Cance W. (2010). Focal adhesion kinase and p53 signal transduction pathways in cancer. Front. Biosci. 15 (3), 901–912. 10.2741/3653 PMC3136041 20515733

[B11] Gong Y. Fan Z. Luo G. Yang C. Huang Q. Fan K. (2019). The role of necroptosis in cancer biology and therapy. Mol. Cancer 18 (1), 100. 10.1186/s12943-019-1029-8 31122251 PMC6532150

[B12] Huang Y. Q. Liang C. H. He L. Tian J. Liang C. S. Chen X. (2016). Development and validation of a radiomics nomogram for preoperative prediction of lymph node metastasis in colorectal cancer. J. Clin. Oncol. 34 (18), 2157–2164. 10.1200/jco.2015.65.9128 27138577

[B13] Huang Y. Zou Y. Xiong Q. Zhang C. Sayagués J. M. Shelat V. G. (2021). Development of a novel necroptosis-associated miRNA risk signature to evaluate the prognosis of colon cancer patients. Ann. Transl. Med. 9 (24), 1800. 10.21037/atm-21-6576 35071494 PMC8756225

[B14] Jia Q. Liao X. Zhang Y. Xu B. Song Y. Bian G. (2022). Anti-tumor role of CAMK2B in remodeling the stromal microenvironment and inhibiting proliferation in papillary renal cell carcinoma. Front. Oncol. 12, 740051. 10.3389/fonc.2022.740051 35127542 PMC8815460

[B15] Jiang W. Agrawal D. K. Boosani C. S. (2018). Cell-specific histone modifications in atherosclerosis (Review). Mol. Med. Rep. 18 (2), 1215–1224. 10.3892/mmr.2018.9142 29901135 PMC6072136

[B16] Kast R. E. Alfieri A. Assi H. I. Burns T. C. Elyamany A. M. Gonzalez-Cao M. (2022). Mdact: A new principle of adjunctive cancer treatment using combinations of multiple repurposed drugs, with an example regimen. Cancers (Basel) 14 (10), 2563. 10.3390/cancers14102563 35626167 PMC9140192

[B17] Kehoe J. Khatri V. P. (2006). Staging and prognosis of colon cancer. Surg. Oncol. Clin. N. Am. 15 (1), 129–146. 10.1016/j.soc.2005.08.006 16389154

[B18] Krysko O. Aaes T. L. Kagan V. E. D'Herde K. Bachert C. Leybaert L. (2017). Necroptotic cell death in anti-cancer therapy. Immunol. Rev. 280 (1), 207–219. 10.1111/imr.12583 29027225

[B19] Lebrec H. Ponce R. Preston B. D. Iles J. Born T. L. Hooper M. (2015). Tumor necrosis factor, tumor necrosis factor inhibition, and cancer risk. Curr. Med. Res. Opin. 31 (3), 557–574. 10.1185/03007995.2015.1011778 25651481

[B20] Li X. Xiang L. Lin Y. Tang Q. Meng F. Chen W. (2022). Computational analysis illustrates the mechanism of qingfei paidu decoction in blocking the transition of COVID-19 patients from mild to severe stage. Curr. Gene Ther. 22 (3), 277–289. 10.2174/1566523221666210907162005 34493195

[B21] Liu L. Huang L. Chen W. Zhang G. Li Y. Wu Y. (2022). Comprehensive analysis of necroptosis-related long noncoding RNA immune infiltration and prediction of prognosis in patients with colon cancer. Front. Mol. Biosci. 9, 811269. 10.3389/fmolb.2022.811269 35237659 PMC8883231

[B22] Liu M. L. Zang F. Zhang S. J. (2019). RBCK1 contributes to chemoresistance and stemness in colorectal cancer (CRC). Biomed. Pharmacother. 118, 109250. 10.1016/j.biopha.2019.109250 31545242

[B23] Liu X. Zhou M. Mei L. Ruan J. Hu Q. Peng J. (2016). Key roles of necroptotic factors in promoting tumor growth. Oncotarget 7 (16), 22219–22233. 10.18632/oncotarget.7924 26959742 PMC5008357

[B24] Liu Z. Guan C. Lu C. Liu Y. Ni R. Xiao M. (2018). High NUSAP1 expression predicts poor prognosis in colon cancer. Pathol. Res. Pract. 214 (7), 968–973. 10.1016/j.prp.2018.05.017 29853313

[B25] Ma J. Song B. Wei Z. Huang D. Zhang Y. Su J. (2022). m5C-Atlas: a comprehensive database for decoding and annotating the 5-methylcytosine (m5C) epitranscriptome. Nucleic Acids Res. 50 (D1), D196–d203. 10.1093/nar/gkab1075 34986603 PMC8728298

[B26] Miller K. D. Nogueira L. Mariotto A. B. Rowland J. H. Yabroff K. R. Alfano C. M. (2019). Cancer treatment and survivorship statistics, 2019. Ca. Cancer J. Clin. 69 (5), 363–385. 10.3322/caac.21565 31184787

[B27] Moon S. W. Son H. J. Choi E. J. Yoo N. J. Lee S. H. (2021). Brief research report regional difference in TRAF2 and TRAF3 gene mutations in colon cancers. Pathol. Oncol. Res. 27, 625438. 10.3389/pore.2021.625438 34257589 PMC8262244

[B28] Morris G. M. Huey R. Lindstrom W. Sanner M. F. Belew R. K. Goodsell D. S. (2009). AutoDock4 and AutoDockTools4: Automated docking with selective receptor flexibility. J. Comput. Chem. 30 (16), 2785–2791. 10.1002/jcc.21256 19399780 PMC2760638

[B29] Nersisyan S. Novosad V. Engibaryan N. Ushkaryov Y. Nikulin S. Tonevitsky A. (2021). ECM-receptor regulatory network and its prognostic role in colorectal cancer. Front. Genet. 12, 782699. 10.3389/fgene.2021.782699 34938324 PMC8685507

[B30] Pei H. J. Yang J. Hu F. X. Chen Y. Z. Yang C. H. (2021). Tribulus terrestris L. protects glomerular endothelial cells via the miR155-H2AC6 interaction network in hypertensive renal injury. Ann. Transl. Med. 9 (21), 1626. 10.21037/atm-21-5641 34926670 PMC8640897

[B31] Qi L. Xu R. Ren X. Zhang W. Yang Z. Tu C. (2022). Comprehensive profiling reveals prognostic and immunogenic characteristics of necroptosis in soft tissue sarcomas. Front. Immunol. 13, 877815. 10.3389/fimmu.2022.877815 35663937 PMC9159500

[B32] Reina S. Guarino F. Magrì A. De Pinto V. (2016). VDAC3 as a potential marker of mitochondrial status is involved in cancer and pathology. Front. Oncol. 6, 264. 10.3389/fonc.2016.00264 28066720 PMC5179545

[B33] Seehawer M. Heinzmann F. D'Artista L. Harbig J. Roux P. F. Hoenicke L. (2018). Necroptosis microenvironment directs lineage commitment in liver cancer. Nature 562 (7725), 69–75. 10.1038/s41586-018-0519-y 30209397 PMC8111790

[B34] Song B. Chen K. Tang Y. Wei Z. Su J. de Magalhães J. P. (2021). ConsRM: Collection and large-scale prediction of the evolutionarily conserved RNA methylation sites, with implications for the functional epitranscriptome. Brief. Bioinform. 22 (6), bbab088. 10.1093/bib/bbab088 33993206

[B35] Song B. Tang Y. Chen K. Wei Z. Rong R. Lu Z. (2020). m7GHub: deciphering the location, regulation and pathogenesis of internal mRNA N7-methylguanosine (m7G) sites in human. Bioinformatics 36 (11), 3528–3536. 10.1093/bioinformatics/btaa178 32163126

[B36] Stoll G. Ma Y. Yang H. Kepp O. Zitvogel L. Kroemer G. (2017). Pro-necrotic molecules impact local immunosurveillance in human breast cancer. Oncoimmunology 6 (4), e1299302. 10.1080/2162402x.2017.1299302 28507808 PMC5414877

[B37] Su Z. Yang Z. Xu Y. Chen Y. Yu Q. (2015). Apoptosis, autophagy, necroptosis, and cancer metastasis. Mol. Cancer 14, 48. 10.1186/s12943-015-0321-5 25743109 PMC4343053

[B38] Subramanian A. Narayan R. Corsello S. M. Peck D. D. Natoli T. E. Lu X. (2017). A next generation connectivity map: L1000 platform and the first 1, 000, 000 profiles. Cell 171 (6), 1437–1452. 10.1016/j.cell.2017.10.049 29195078 PMC5990023

[B39] Szklarczyk D. Santos A. von Mering C. Jensen L. J. Bork P. Kuhn M. (2016). Stitch 5: Augmenting protein-chemical interaction networks with tissue and affinity data. Nucleic Acids Res. 44 (D1), D380–D384. 10.1093/nar/gkv1277 26590256 PMC4702904

[B40] Tang Y. Chen K. Song B. Ma J. Wu X. Xu Q. (2021). m6A-Atlas: a comprehensive knowledgebase for unraveling the N6-methyladenosine (m6A) epitranscriptome. Nucleic Acids Res. 49 (D1), D134–d143. 10.1093/nar/gkaa692 32821938 PMC7779050

[B41] Torrens-Mas M. Alorda-Clara M. Martínez-Vigara M. Roca P. Sastre-Serra J. Oliver J. (2022). Xanthohumol reduces inflammation and cell metabolism in HT29 primary colon cancer cells. Int. J. Food Sci. Nutr. 73 (4), 471–479. 10.1080/09637486.2021.2012561 34879764

[B42] Trott O. Olson A. J. (2010). AutoDock Vina: Improving the speed and accuracy of docking with a new scoring function, efficient optimization, and multithreading. J. Comput. Chem. 31 (2), 455–461. 10.1002/jcc.21334 19499576 PMC3041641

[B43] Wang R. Li H. Wu J. Cai Z. Y. Li B. Ni H. (2020). Gut stem cell necroptosis by genome instability triggers bowel inflammation. Nature 580 (7803), 386–390. 10.1038/s41586-020-2127-x 32296174

[B44] Wang Y. Zhao M. He S. Luo Y. Zhao Y. Cheng J. (2019). Necroptosis regulates tumor repopulation after radiotherapy via RIP1/RIP3/MLKL/JNK/IL8 pathway. J. Exp. Clin. Cancer Res. 38 (1), 461. 10.1186/s13046-019-1423-5 31706322 PMC6842489

[B45] Wei J. Hu M. Huang K. Lin S. Du H. (2020). Roles of proteoglycans and glycosaminoglycans in cancer development and progression. Int. J. Mol. Sci. 21 (17), E5983. 10.3390/ijms21175983 32825245 PMC7504257

[B46] Wu L. Zhang X. Zheng L. Zhao H. Yan G. Zhang Q. (2020). RIPK3 orchestrates fatty acid metabolism in tumor-associated macrophages and hepatocarcinogenesis. Cancer Immunol. Res. 8 (5), 710–721. 10.1158/2326-6066.cir-19-0261 32122992

[B47] Yan J. Wan P. Choksi S. Liu Z. G. (2022). Necroptosis and tumor progression. Trends Cancer 8 (1), 21–27. 10.1016/j.trecan.2021.09.003 34627742 PMC8702466

[B48] Yang Z. Lu S. Wang Y. Tang H. Wang B. Sun X. (2022). A novel defined necroptosis-related miRNAs signature for predicting the prognosis of colon cancer. Int. J. Gen. Med. 15, 555–565. 10.2147/ijgm.s349624 35046713 PMC8763259

[B49] Yerra V. G. Advani A. (2018). Histones and heart failure in diabetes. Cell. Mol. Life Sci. 75 (17), 3193–3213. 10.1007/s00018-018-2857-1 29934664 PMC6063320

[B50] Yu G. Wang L. G. Han Y. He Q. Y. (2012). clusterProfiler: an R package for comparing biological themes among gene clusters. Omics 16 (5), 284–287. 10.1089/omi.2011.0118 22455463 PMC3339379

