# Supplementary material for: Development of necroptosis-related gene signature to predict the prognosis of colon adenocarcinoma
Source: Front Genet. 2022 Oct 24;13:1051800. doi: 10.3389/fgene.2022.1051800 (PMC9639779; doi:10.3389/fgene.2022.1051800)
Supplement: Supplementary file 1 [file DataSheet1.docx]

**Supplementary Materials**

**Supplementary Figures**


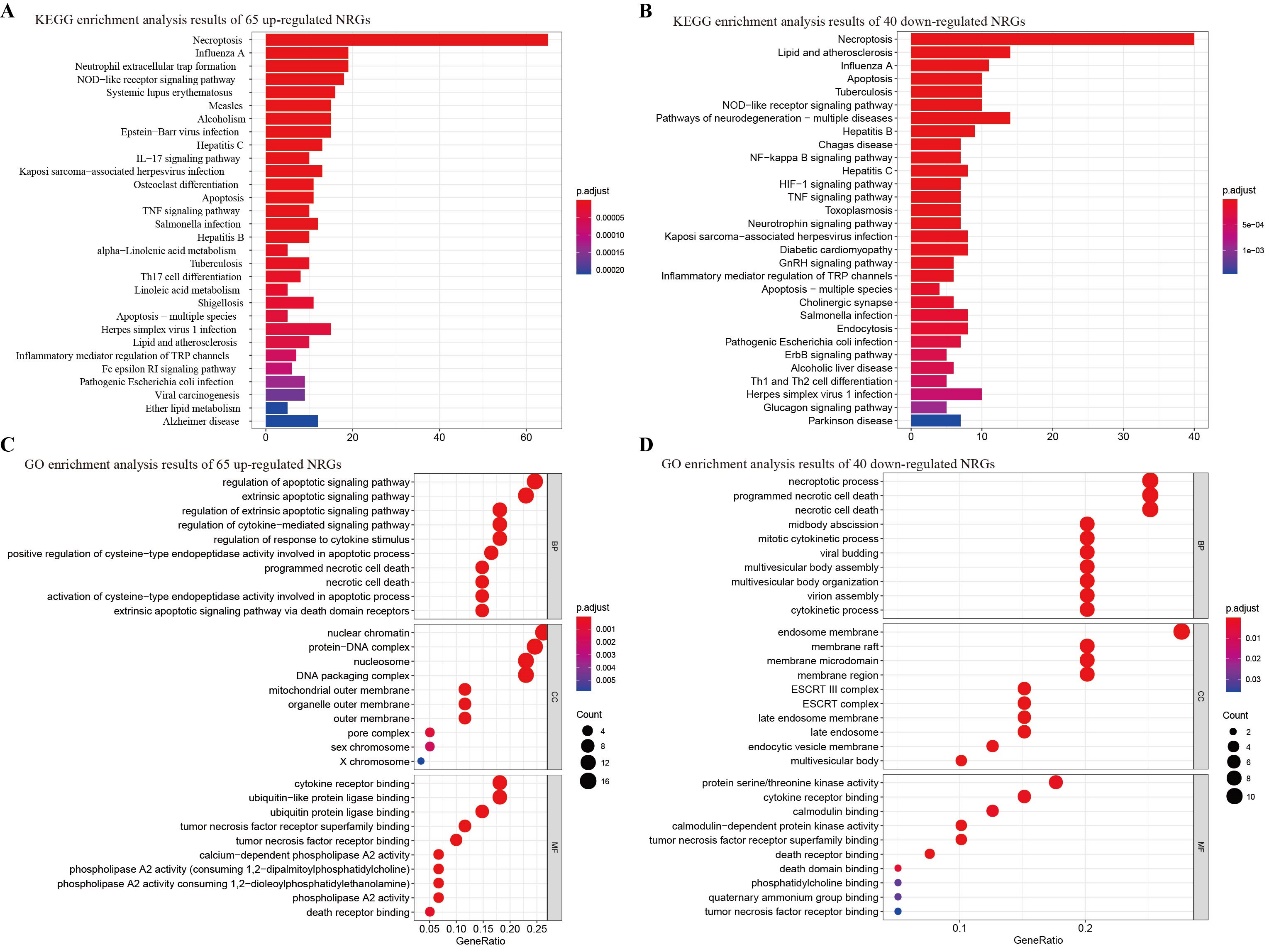


**Figure S1** (A) KEGG enrichment analysis results of 65 up-regulated NRGs; (B) KEGG enrichment analysis results of 40 down-regulated NRGs; (C) GO enrichment analysis results of 65 up-regulated NRGs; (D) GO enrichment analysis results of 40 down-regulated NRGs.


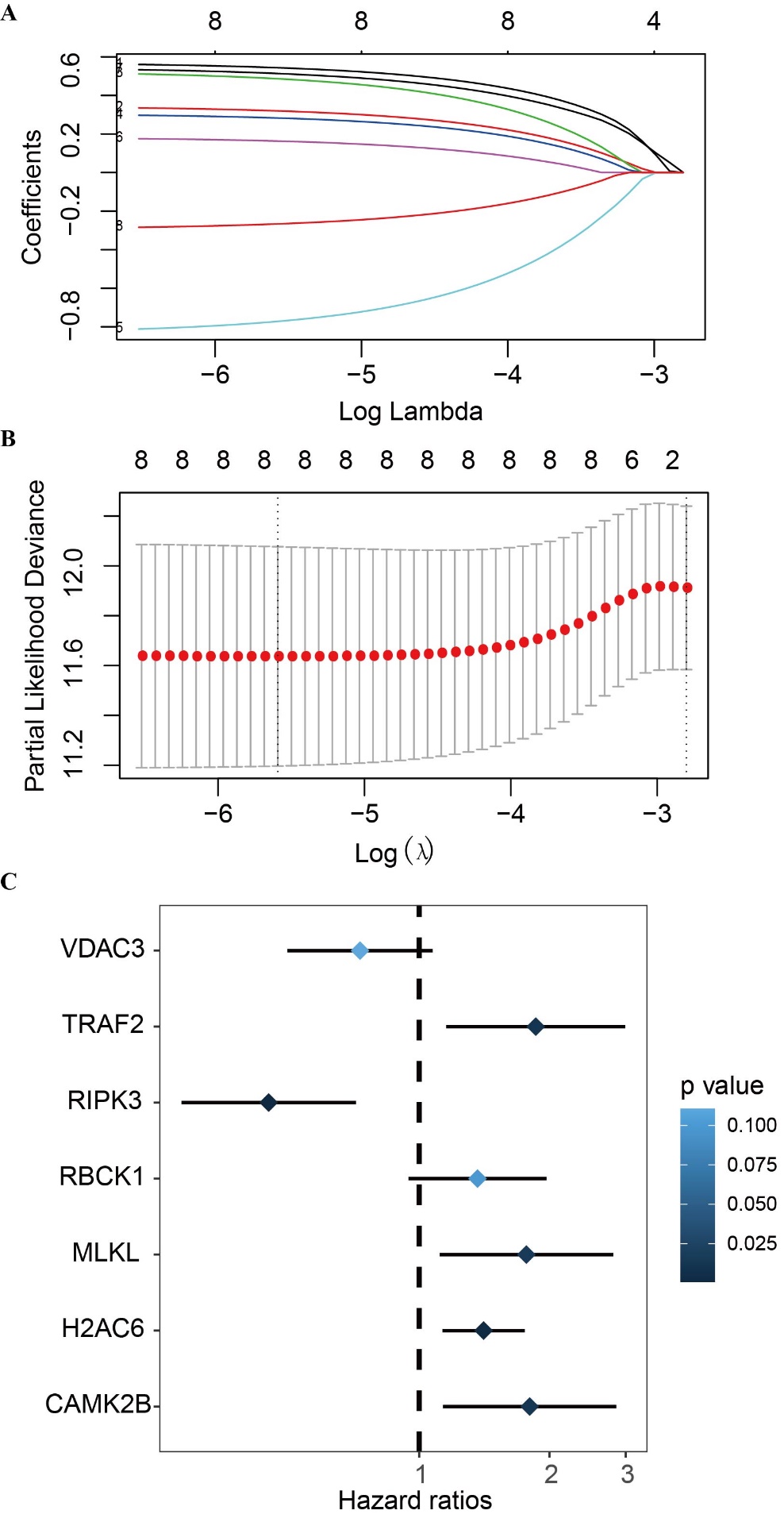


**Figure S2** (A-B) LASSO regression. (C) The forest map of prognostic gene.


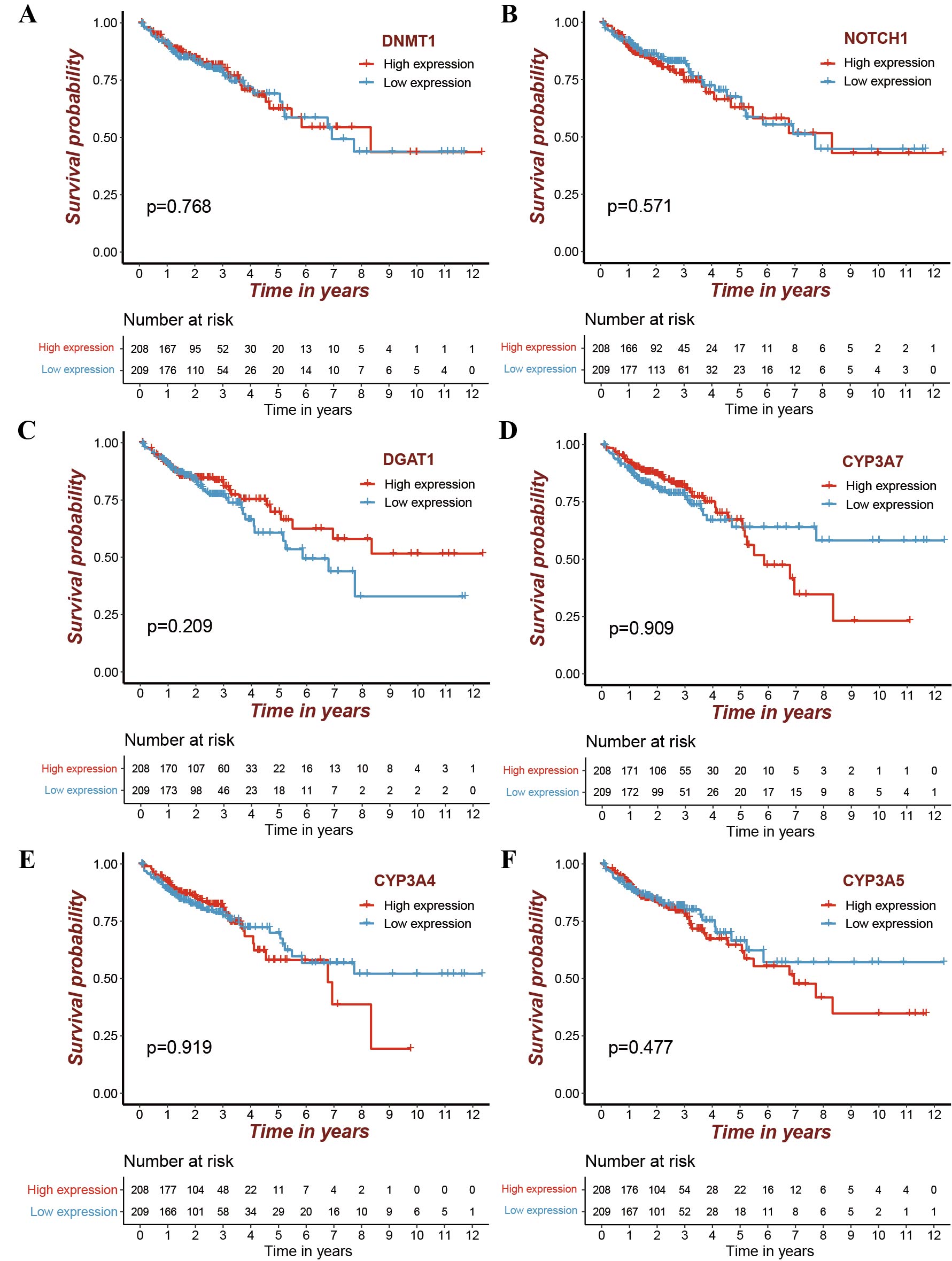


**Figure S3** CYP3A4, NOTCH1, DNMT1, DGAT1, CYP3A5 and CYP3A7 are not significant for predicting the survival rate of COAD patients.

**Supplementary Tables**

**Supplementary Table S1** 159 necroptosis-related genes used in this study.

**Supplementary Table S2** Summary of differential expression genes (DEGs) between tumor and normal samples in TCGA cohort.

**Supplementary Table S3** Summary of univariate Cox regression analyses of necroptosis related genes in TCGA cohort.

**Supplementary Table S4** Summary of multivariate Cox regression analyses of necroptosis related genes in TCGA cohort.

**Supplementary Table S5** The results of GSEA analysis.

**Supplementary Table S6** The 237 differently expressed gens in high risk group.

**Supplementary Table S7** The outputs from the Cmap database.
